# Engineering Abiotic Stress Tolerance in Crop Plants through CRISPR Genome Editing

**DOI:** 10.3390/cells11223590

**Published:** 2022-11-13

**Authors:** Mehboob-ur Rahman, Sana Zulfiqar, Muhammad Ahmad Raza, Niaz Ahmad, Baohong Zhang

**Affiliations:** 1 Plant Genomics and Molecular Breeding Laboratory, National Institute for Biotechnology and Genetic Engineering College, Pakistan Institute of Engineering and Applied Sciences (NIBGE-C, PIEAS), Faisalabad 38000, Pakistan; 2 Department of Biology, East Carolina University, Greenville, NC 27858, USA

**Keywords:** CRISPR, drought, salinity, heat, heavy metals, field crops, sustainable agriculture

## Abstract

Environmental abiotic stresses challenge food security by depressing crop yields often exceeding 50% of their annual production. Different methods, including conventional as well as genomic-assisted breeding, mutagenesis, and genetic engineering have been utilized to enhance stress resilience in several crop species. Plant breeding has been partly successful in developing crop varieties against abiotic stresses owning to the complex genetics of the traits as well as the narrow genetic base in the germplasm. Irrespective of the fact that genetic engineering can transfer gene(s) from any organism(s), transgenic crops have become controversial mainly due to the potential risk of transgene-outcrossing. Consequently, the cultivation of transgenic crops is banned in certain countries, particularly in European countries. In this scenario, the discovery of the CRISPR tool provides a platform for producing transgene-free genetically edited plants—similar to the mutagenized crops that are not extensively regulated such as genetically modified organisms (GMOs). Thus, the genome-edited plants without a transgene would likely go into the field without any restriction. Here, we focused on the deployment of CRISPR for the successful development of abiotic stress-tolerant crop plants for sustaining crop productivity under changing environments.

## 1. Introduction

The process of crop domestication, which started at the dawn of human civilization, has led to the development of high-yielding crop varieties. These crop varieties have played a significant role in the transformation of every aspect of human society. However, these crops cannot withstand the changing environmental conditions and therefore these crops are undergoing significant yield losses. This phenomenon has recently been witnessed in Pakistan where the early onset of high-temperature regimes followed by heavy rains have almost entirely damaged the crops, especially rice and cotton. The rainfall has not only affected the standing crops but its knockdown effects are also extending this cropping season as well. For instance, it is feared that the next crop—wheat, a staple food crop in the country—would not be sown in many flooded regions in this growing season. The other abiotic stresses including drought, salinity, temperature, and heavy metal toxicity [1] are also gradually converting the cultivated lands into barren soils. Thus, all abiotic stresses in the face of changing climatic conditions are threatening global food security and are a major obstacle in realizing the UN’s target of a 70–100% increase in crop productivity by 2050.

Abiotic stresses can result in significant yield losses. For instance, drought alone can cause a 50–70% reduction in crop yield for different crops [2]. For example, due to drought stress, 40% yield losses were reported in maize [3], 21% in wheat [3], 50% in rice [4], 27–40% in chickpea [5], 42% in soybean [6], and 68% in cowpea [7]. Salinity stress is the second most devastating menace that not only reduces crop productivity but also deteriorates fertile lands [8]. About one-fifth of the agricultural irrigated land is affected by excessive salts [9,10]. Poor quality irrigation water together with changing climatic conditions and the excessive use of chemicals including fertilizers and pesticides continue to add new acreage under salinity stress. It was estimated that the excessive use of chemicals including fertilizers and pesticides will cause 50% of the cultivated lands to be saline by 2050 [11]. The third most important factor is the increase in temperature that may depress crop production substantially. For example, every 1 °C increase in atmospheric temperature reduces wheat yield by 6% [12], rice yield by 10–20% [13], and 21–31% corn yield [14]. This means that developing stress-tolerant crops could increase the crop yield. It has been a widely accepted fact that developing resilient crop varieties that can withstand the impact of abiotic stresses including changing climatic conditions is the only option for harvesting sustainable crop productions. 

Although conventional breeding as well as molecular techniques and genetic engineering contributed significantly to producing biotic stress-resilient crop varieties [15], however, limited success was achieved in addressing abiotic stresses owing to the complex genetics involved in resistance mechanisms. New genetic tools including genome editing are being used extensively for developing resilient crops but stringent regulations for cultivating such crops remained a major hurdle in the spread of genome-edited crops [16,17]. Improvements in the genome editing assay including clustered regularly interspaced short palindromic repeat (CRISPR) have caused it to be possible to edit the genome very precisely [18,19,20]. Genome editing can be used for studying novel traits and targeting the improvement of traits for mitigating the impact of abiotic stresses [21,22]. In this review, we will discuss the role of CRISPR-Cas9 in the development of stress-resilient crops for addressing nutrient use efficiency, drought, salinity, temperature, tolerance to environmental pollutants, and heavy metal toxicity. 

## 2. Genome Editing Machinery

Genome editing tools use a special class of nucleases that can modify a specific nucleotide(s) in the genome(s) by introducing target-specific double-stranded breaks. Four different types of nucleases including meganucleases (MegNs), zinc finger nucleases (ZFNs), transcription activator-like effector nucleases (TALENs), and clustered regularly interspaced short palindromic repeats (CRISPR)/associated proteins (Cas) [23] have been reported [1]. Meganucleases, ZFNs, and TALENs can induce DSBs at the target site by DNA-protein interaction. However, their utility in genome editing is relatively laborious and time-consuming [24]. On the other hand, the deployment of CRISPR for editing genomes is relatively easy, efficient, and economical [25]. CRISPR-Cas9 was derived from the bacterial immune defense that targets the invading viruses [26,27]. Being a single effector molecule system, CRISPR/Cas9 belongs to class 2-type II and is by far the most widely used system for editing genomes precisely [28,29]. 

**Figure 1:**
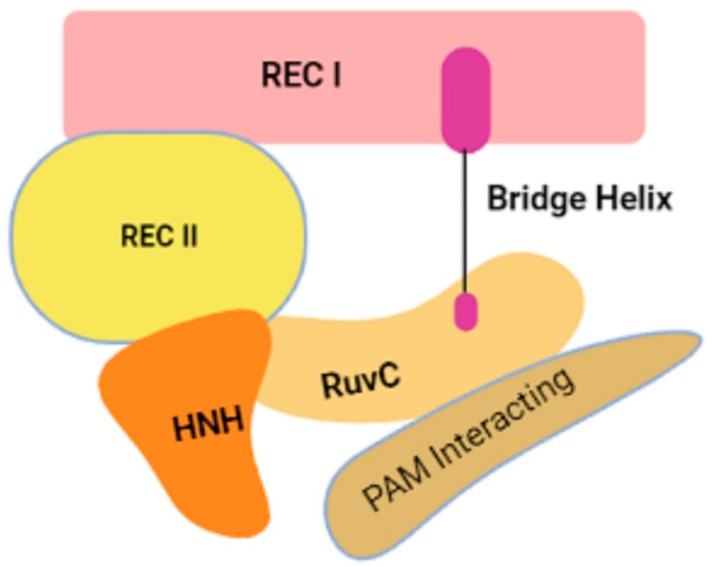
The structure of Cas9 protein—the major component of the CRISPR/Cas9 system.

The CRISPR/Cas9 comprises Cas9 and single guide RNA (sgRNA). The sgRNA is categorized into two components viz. CRISPR-RNA (crRNA) and trans-activating RNA (tracrRNA). The crRNA is a 20-nucleotide-long complementary sequence to target the sequence of interest, pre-crRNA, which joins with tracrRNA to produce a double-stranded RNA [30]. The RNase III activates the pre-crRNA for converting to mature crRNA [31]. The Cas9 nuclease has six domains: two recognition domains (REC I and REC II) essential for binding sgRNA and DNA, two nuclease domains (HNH and RuvC) for the cleavage of the complementary and non-complementary strands at the target region, the protospacer adjacent motif (PAM) interaction (PI) domain, and the bridge helix domain for the initiation of nuclease activity (Figure 1) [32,33]. 

The Cas enzymes had many orthologues that recognize different and specific PAM sequences (Table 1). For example, *Streptococcus pyogene* Cas9 (spCas9) recognizes the NGG sequence where N could be any nucleotide [30,34,35,36]. Different Cas proteins require the PAM position at the different directions of the target sequence. Certain Cas proteins require PAM at 5′ (such as Cas12), while the majority of the Cas require PAM at the 3′ site of the target DNA.

**Table 1:** Different CRISPR/Cas systems, classes, and types.

Name	Cas	Organism	Type	PAM *	PAM Location	References
SpCas9	Cas9	Streptococcus pyogenes	Type II	NGG	3′	[30]
SaCas9	Cas9	Streptococcus aureus	Type II	NNGRRT	3′	[37,38,39]
FnCas9	Cas9	Francisella Novicida	Type II	NGG	3′	[40]
NmCas9	Cas9	Neisseria meningitidis	Type II	NNNNGATT	3′	[41]
CjCas9	Cas9	Campylobacter jejuni	Type II	NNNNRYAC and NNNNACAC	3′	[42]
St1Cas9	Cas9	Streptococcus thermophilus	Type II	NNAGAAW	3′	[38,43]
St3Cas9	Cas9	Streptococcus thermophilus	Type II	NGGNG	3′	[29]
AsCas12a	Cas12a(cpf1)	Acidaminococcus sp.	Type V	TTTV	5′	[44,45]
LbCas12a	Cas12a(cpf1)	Lachnospiraceae bacterium	Type V	TTTV	5′	[45,46]
FnCas12a	Cas12a(cpf1)	Francisella Novicida	Type V	TTTN or YTN	5′	[45]
LsCas13 **	Cas13(C2c2)	Leptotrichia shahii	Type VI	Non g nucleotide at the 3′ protospacer flanking site	3′	[47]
Cas14 ***	Cas14	Archea	Type V	Thymine rich PAM sequences	3′	[48]
FnCas9 variant	Cas9	Modified FnCas9	Type II	YG	3′	[49]
SpCas9-VQR	Cas9	Engineered SpCas9	Type II	NGA	3′	[50,51,52]
SpCas9-EQR	Cas9	Engineered SpCas9	Type II	NGAG	3′	[50,51,52]
SpCas9-NG	Cas9	Engineered SpCas9	Type II	NG	3′	[53,54,55]
SpCas9-VRER	Cas9	Engineered SpCas9	Type II	NGCG	3′	[52]
GeoCas9		Geobacillus stearothermophilus	Type II	NNNNCRAA	3′	[48]
SaCas9-KKH	Cas9	Engineered SaCas9	Type II	NNNRRT	3′	[56]
SpCas9-HF	Cas9	Engineered SpCas9	Type II	NGG	3′	[57,58]
eSpCas9	Cas9	Engineered SpCas9	Type II	NGG	3′	[57,58]
xCas9	Cas9	Engineered SpCas9	Type II	NG, GAA and GAT	3′	[54]
Sniper-Cas9	Cas9	Engineered SpCas9	Type II	NGG	3′	[59]
evoCas9	Cas9	Mutated SpCas9	Type II	NGG	3′	[60]
HypaCas9	Cas9	Mutated SPCas9-HF	Type II	NGG	3′	[61]
Cas9-NRNH	Cas9	Engineered SpCas9	Type II	NRNH	3′	[62]
SpG	Cas9	Engineered SpCas9	Type II	NGN	3′	[63]
SpRY	Cas9	Engineered SpCas9	Type II	NRN or NYN	3′	[62,64]
ScCas9	Cas9	Streptococcus canis	Type II	NNG	3′	[65]
LbCas12a-RR	Cas12	Engineered LbCas12a	Type V	TYCV, CCCC	5′	[66,67]
LbCas12a-RVR	Cas12	Engineered LbCas12a	Type V	TATV	5′	[66,67]
FnCas12a-RVR	Cas12	Engineered FnCas12a	Type V	TATG	5′	[66]
enLbCas12a	Cas12	Engineered LbCas12a	Type V	TTTV	5′	[46]
ttLbCas12a	Cas12	Engineered LbCas12a	Type V	TTTV	5′	[46,68]
AacCas12b	Cas12	Alicyclobacillus acidoterrestris	Type V	VTTV	5′	[65,69]
AaCas12b	Cas12	Acidaminococcus sp.	Type V	VTTV	5′	[69]
BthCas12b	Cas12	*Bacillus thermoamylovorans*	Type V	ATTN	5′	[69]
BhCas12b v4	Cas12	*Bacillus hisashii*	Type V	ATTN	5′	[70]
BvCas12b	Cas12	Engineered Cas12a	Type V	ATTN	5′	[71]
Lb5Cas12a	Cas12	Engineered LbCas12a	Type V	TTTV	5′	[72]
BsCas12a	Cas12	Engineered Cas12a	Type V	TTTV	5′	[72]
Mb2Cas12a	Cas12	Engineered Cas12a	Type V	TTV	5′	[72]
TsCas12a	Cas12	Thiomicrospira sp.	Type V	TTTV	5′	[72]
MCas12a	Cas12	Engineered Cas12a	Type V	TTTV	5′	[72]
BoCas12a	Cas12	Engineered Cas12a	Type V	TTTV	5′	[72]
MbCas12a	Cas12	Engineered Cas12a	Type V	TTTV	5′	[72]
Mb2Cas12a-RVR	Cas12	Engineered Cas12a	Type V	TATV	5′	[72]
Mb2Cas12a-RVR	Cas12	Engineered Cas12a	Type V	TTTTV, TTV, TATV, TYCV, CCCV, CTCV	5′	[72]

* “N” represents any nucleotide. “R” represents A or G. “H” represents A, C, or T. “Y” represents C or T. “W” represents A or T in PAM sequence. ** Cas13 targets RNA sequences. *** Cas14 targets the single stranded DNA sequence that is why it does not require PAM.

**Figure 2:**
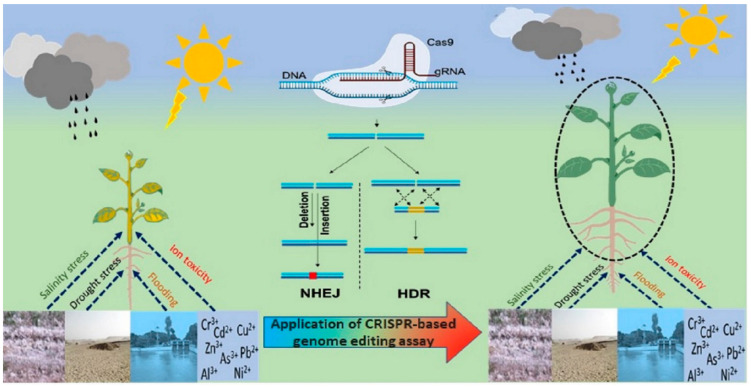
The schematic illustration of the CRISPR-based genome editing for improvement in resilience against various abiotic stresses.

The Cas9 endonuclease is activated upon binding with mature gRNA that induces conformational changes to it. Upon binding with target DNA, Cas induces double-stranded break 3 nucleotide upstream to the PAM sequence (5′ NGG 3′). These breaks will be repaired either through non-homologous end joining (NHEJ) or the homologous directed repair (HDR) pathway [73], depending on the presence of the DNA template with homology to the flanking position of the DSB. During the cut and repair mechanism, some nucleotides are removed or added to the original sequence, which may alter the protein structure or completely abolish its function [74]. Genome editing can knock out or overexpress an individual gene based on different repair mechanisms (Figure 2) [75,76,77,78]. Several studies have shown the utility of genome editing assays especially CRISPR/Cas9 for improving tolerance to various stresses including drought, salinity, heat, heavy metals, etc. (Table 2).

**Table 2:** Crop improvement with tolerance to abiotic stress by using CRISPR genome editing.

Crop Species	Targeted Gene	Function	Phenotype	References
Rice	*DERF1, PMS3, MSH1, MYB5, SPP*	Amino acid synthesis and drought tolerance	DT	[79]
Rice	*SRL1, SRL2*	Regulate leaf rolling	DT	[80]
Rice	*ERA1*	Regulates ABA signaling and dehydration response	DT	[81]
Tomato	*GID1a*	Gibberellin (GA) receptor	DT	[82]
Tomato	*LBD40*	Involved in jasmonic acid (JA)-mediated stress response	DT	[83]
Maize	*ARGOS8*	Involved in ethylene response	DT	[84]
Maize	*abh2*	Abscisic acid 8′-hydroxylase mediates stomatal opening	DT	[85]
Rapeseed	*A6.RGA*	DELLA protein, negative regulator of gibberellin signaling	DT	[86]
Maize	*STL1*	Dirigent protein localized to the Casparian strip	ST	[87]
Tomato	*ABIG1*	Homeodomain-leucine Zipper (HD-ZIP) TF	ST	[88]
Tomato	*HyPRP1*	Negative regulator of salt stress	ST	[89]
Soybean	*AITR*	Regulation of ABA signaling	ST	[90]
Rice	*SPL10*	Regulate trichome development	ST	[91]
Rice	*RAV2*	Function in the regulation of developmental processes	ST	[92]
Rice	*RR9, RR10*	Negatively regulate cytokinin signaling	ST	[93]
Rice	*DST*	Involved in stomata development	ST	[94]
Rice	*SOS1*	Na+/H+ antiporter mediating Na+ transport	ST	[95]
Rice	*GI*	Circadian clock component	ST	[96]
Rice	*bHLH024*	Basic helix–loop–helix TF involved in growth and stress responses	ST	[97]
Rice	*RR22*	Involved in cytokinin signaling	ST	[98]
Rice	*PQT3*	E3 ubiquitin ligase	ST	[99]
Rice	*miR535*	Involved in Salinity stress regulation	ST	[100]
Rice	*HSA1*	Chloroplast development and protection	HT	[101]
Tomato	*MAPK3*	Negative regulator of heat stress	HT	[102]
Rice	*PYL1* *PYL4* *PYL6*	Regulatory component of Abscisic acid	HT	[103]
Tomato	*AGL6*	Involved in fruit development	HT	[21]
Rice	*MYB30*	Negative regulator of cold stress	CT	[104]
Rice	*Nramp5*	Role in Cadmium translocation	HMT	[105]
Rice	*HAK1*	Transportation of Cesium	HMT	[106]
Rice	*ARM1*	Regulation of Arsenic response	HMT	[107]
Rice	*ALS*	Involved in herbicide tolerance	HerT	[108,109,110]
Watermelon	*ALS*	Involved in herbicide tolerance	HerT	[111]
Maize	*ALS1* *ALS2*	Involved in herbicide tolerance	HerT	[112]

“DT” Drought tolerance, “ST” Salinity tolerance, “HT” Heat tolerance, “CT” Cold tolerance, “HMT” Heavy metal tolerance, “HerT” Herbicide Tolerance.

## 3. CRISPR for Improving Drought Tolerance in Crop Plants

Drought, aggravated by climate change effects such as uneven rainfall patterns and increasing temperature, is becoming a threat to sustainable agriculture in many parts of the world. Tolerance to drought stress is a complex quantitative trait that is attributed to multiple physiological and biochemical processes [113]. Many efforts were performed to tailor these genes as well as to add new genes by adopting transgenic approaches. The transgenic crops were not commercialized due to the marginal impact of the transgene(s) in conferring drought tolerance and strict regulatory policies for the release of GM crops in the environment. After the discovery of genome editing tools, experiments are being designed to edit the genes involved in drought tolerance pathways for increasing the public acceptance of genome-edited crops [114]. Many studies have reported the conferring of drought tolerance in plants through CRISPR. For instance, downregulating the expression of *DERF1*, *PMS3*, *MSH1*, *MYB5*, and *SPP* regulatory genes using CRISPR/Cas9 have shown to result in drought tolerance enhanced in rice [79]. Mutation induced in Arabidopsis *OST2* structural gene through deploying CRISPR/Cas9 demonstrated drought tolerance [115]. 

In another study, CRISPR/Cas9-mediated knockout of the *miR169a* gene in Arabidopsis resulted in a significant improvement of drought tolerance [116]. Similarly, activation of the vacuolar H^+^-pyrophosphate (*AVP1*) regulatory gene with CRISPR/Cas9 resulted in drought tolerance improving in Arabidopsis [117]. Likewise, activation of the abscisic acid-responsive element binding gene (*AREB1*) by CRISPR/Cas9a exhibited enhanced drought tolerance in Arabidopsis [118]. Recently, the silencing of the trehalase (*TRE1*) gene through CRISPR/Cas9 demonstrated drought tolerance in *Arabidopsis thaliana* [119]. Also, the editing in the *STL1* structural gene conferred improved drought tolerance in *A. thaliana*. In maize, editing of the *ARGOS8* gene—the negative regulator of ethylene response—through CRISPR/Cas9 enhanced drought tolerance [84]. Also, suppressing the expression of the abscisic acid hydroxylase 2 (*abh2*) gene improved drought tolerance in maize [85]. Among oil seed crops, CRISPR/Cas9 was deployed to edit the *A6.RGA* gene, which showed significant enhancement in drought tolerance in rapeseed [86]. In rice, the CRISPR/Cas9-mediated knockout of *SRL1*, *SRL2*, and *ERA1* genes improved drought tolerance [80,81]. Multiple genes were also edited in tomato plants through CRISPR/Cas9 assay for improving drought tolerance. For instance, the gibberellin insensitive dwarf1 (*GID1*) gene [82] and the LBD40 gene were edited [83]. In wheat, the *SAL1* gene negative regulator of drought tolerance was edited through multiplex CRISPR/Cas9 assay that improves drought tolerance at the seedling stage [120]. Cotton’s ability to withstand drought can be improved by CRISPR/Cas genome editing of the *HB12* gene [121]. 

## 4. CRISPR for Improving Salt Tolerance in Crop Plants 

To meet the increasing world food demand, the UN has estimated that 70–100% of crop production should be increased by the end of 2050. However, at the same time, increasing crop cultivation has led to reduced soil fertility and salinization, which are quite unsuitable for crop growth and cultivation. Soil salinization occurs due to the accumulation of excessive soluble salts in the crop root zone, which hinders water absorption by roots. Consequently, plants exhibit osmotic stress along with nutritional imbalance that pose detrimental effects on plant morphology, plant biochemistry, and biomass and ultimately result in irreversible damage to plants [122,123,124]. Salt stress/salinity also increases the level of reactive oxygen species (ROS); resultantly, the cellular as well as metabolic activities of plants are badly affected [125,126]. The toxic impact of ROS is lipid peroxidation and membrane deterioration, as well as DNA and protein damage [127]. Salt stress hinders the photosynthetic machinery and transpiration by reducing chlorophyll content and stomatal conductance and impairing the chloroplast and photosystem II development [128]. In addition, it lowers the soil and leaf water potential; reduces plant turgor pressure by affecting water relations and ends up with osmotic stress [129]. Consequently, plants suffer a reduced leaf area, reduced photosynthesis, less production of biomass, poor seed germination, and reduced crop yield [130,131,132]. 

Salinity tolerance is conferred by a series of molecular as well as physiological mechanisms in plants [133]. Genome editing and genetic engineering tools have been deployed to target genes involved in ion transport for regulating osmotic adjustment under salt stress [134]. The overexpression of *SOS1* (salt overly sensitive 1) increased the salinity tolerance in Arabidopsis [135]. Similar to SOS1, overexpression of HvHKT2;1 (subfamily II HKT transporter from Hordeum vulgare) led to increased translocation of Na+, which resulted in enhanced salinity tolerance in barley [136]. In another study, editing in the *OsRR22* gene encoding response regulator (type-B) expressed high tolerance to salinity in rice [98]. In addition, the PARAQUAT TOLERANCE 3 mutants (*OsPQT3*) developed through CRISPR/Cas9 conferred a high degree of salinity tolerance in rice [99]. The role of *OsmiR535* in salt stress tolerance was explored by deploying genome editing tools and it was suggested that the knockout of *OsmiR535* through CRISPR/Cas9 could improve salinity tolerance in rice. Moreover, a homozygous five bp deletion in the coding sequence of OsmiR535 could serve as a potential target for improving salinity tolerance in rice [100]. Another study demonstrated the potential application of CRISPR/Cas9 by manipulating the hybrid proline-rich protein 1 (*HyPRP1*) gene—a negative regulator of salt stress in tomato. The knockdown of *SlHyPRP1* negative-response domain(s) enhanced salinity tolerance at seedling as well as vegetative stages in tomato plants [89]. A gene cluster containing (ACQOS; AT5G46520) and (NLRs; AT5G46510) is involved in osmotic stress tolerance. The role of *ACQOS* was investigated by inducing small insertion/deletion mutations through CRISPR-Cas9, which suggested that *ACQOS* was linked with salt stress resistance directly in Arabidopsis [137]. Although, limited reports are available on the potential implications of the CRISPR/Cas9 system toward the enhancement of salinity tolerance, there is no doubt that the CRISPR/Cas system is a promising tool in improving salinity tolerance in different crops.

## 5. CRISPR/Cas9 for Mitigating the Impact of Heat Stress

The optimum temperature for plant growth and development is 15–24 °C [138,139,140]. Heat stress is explained as the 10–15 °C increase in temperature above the ambient temperature, which is required for normal growth and development. High heat stress emerged as a serious issue responsible for huge yield losses and is expected to exacerbate in the future [141]. Heat stress poses extremely negative effects on plants during all growth stages, from germination to harvesting [142,143]. Heat stress not only aggravates the mortality rate of plants but also deteriorates their quality [144,145]. Plants restrict their growth, metabolism, and cellular activities above the normal temperature. Heat stress also affects the plant’s phenology including its photosynthetic machinery, respiration, and sink/source machinery and impairs photosystem II resulting in reduced production [146,147,148].

Prolonged exposures to extreme temperatures may lead to irreversible changes in plants such as cellular destruction. Plants respond to heat stress by wilting, fruit senescence, bolting, and leaf damage [149]. It causes several molecular, biochemical, and physiological changes that can adversely affect plant growth and productivity and may result in visual symptoms including leaf burn and discoloration [150]. Mostly, reproductive growth was highly affected under heat stress as temperatures ≥ 30 °C may lead to pollen shedding, poor pollen viability, poor germination, and growth of pollen [151]. 

Heat stress affects the physiological processes of plants in several ways. It increases the membrane fluidity, leading to a series of reactions that alter metabolisms and impair cellular machinery [152]. Furthermore, other cellular processes such as protein degradation and cytoskeleton are also influenced by heat stress [153]. Climate change, prolonged heat waves, and global warming are among the leading cause of heat stress [154]. Heat stress severely limits the productivity of crop plants. For instance, it is speculated that each one-degree rise in temperature reduces wheat production by more than 6% [12,155]. Therefore, strategies to mitigate the devastating effects of heat stress are urgently required as global warming is worsening day by day. 

The deletion of heat sensitive albino1 (*OsHSA1*) gene in rice exhibited more sensitivity to heat but had a faster greening phenotype as compared to the wild type. It was demonstrated that HSA1 plays important roles in chloroplast development at early stages and functions in protecting chloroplasts under heat stress at later stages [101]. The *OsHSA1* encodes a fructokinase-like protein that is involved in chloroplast protection and development during different growth stages in rice. Mutants generated through CRISPR/Cas9 in tomato *Slcpk28* showed an increased accumulation of ROS and protein oxidation and decreased the activity of antioxidant enzymes including ascorbate peroxidase under heat stress [156]. The CRISPR/Cas9-mediated knockout of *SlMAPK3* expressed improved heat stress tolerance by decreasing ROS accumulation and up-regulating the expression of genes encoding heat shock proteins (HSPs) and heat stress transcription factors (HSFs) [102].

Brassinosteroids (BRs) are plant hormones involved in conferring tolerance to abiotic stresses in plants [157]. In tomato plants, the *BZR1* gene serves as a key regulator of the BR response. Heat-stress-induced damage was exacerbated in the Δbzr1 mutants and BR-induced heat stress tolerance was lost through the respiratory burst of oxidase homolog (RBOH1)-dependent ROS signaling, which is regulated by feronia homologs [158]. The knockout of *OsNAC006* by CRISPR/Cas9 exhibited an increased level of H_2_O_2_ and superoxide radicals (O_2_–) as well as decreased chlorophyll content and antioxidant enzymes. It indicates that Osnac006 may be involved in heat stress tolerance by mediating the process of photosynthesis and limiting the activity of antioxidant enzymes, triggered in response to oxidative stress under high temperatures [159]. The gene knock-out by CRISPR/Cas9 in genes encoding the abscisic acid receptor (PYL1/4/6) has shown considerable high-temperature tolerance in rice [103]. To address the issues such as global warming and climate change, the identification of targets for improving heat stress tolerance as well as the development of heat tolerant varieties are necessary. Phytochrome (PHY) could be an important target in this respect as PHYB has been identified as a thermo-sensor [160,161]. The PHY mutants expressed improved tolerance to high temperatures in Arabidopsis [162] and tomato plants [163]. This mutant information is important for the definition of targets of genome editing. 

High temperatures result in higher respiration in pollen grains, which can lead to the elimination of respiratory substrates and decreased mitochondrial activity, ultimately resulting in pollen abortion and poor fruit setting [164,165]. Parthenocarpy (fruit development without pollination/fertilization) is an important target for seedless fruit development due to its fertilization independence, consumers’ preference, and good quality of fruit [166,167]. During the screening of an ethyl-methanesulfonate (EMS) mutated population in tomato under heat stress, a mutant capable of generating high-quality seedless fruit was selected. Following the CRISPR/Cas9 gene knockout revealed that the seedless phenotype was caused by a mutation in the tomato *SlAGAMOUSLIKE* 6 (*SlAGL6*) gene encoding MADS-box. Hence, mutations in *SlAGL6* increased heat stress in tomato plants. Moreover, these mutants exhibited facultative parthenocarpy without any pleiotropic effect, which was comparable in both shape and weight to the wild-type fruits (seeded) [21]. Aux/IAA9 (*IAA9*) is responsible for fruit development in tomato and represses parthenocarpy. CRISPR/Cas9-mediated mutant plants showed fruit development without fertilization and mutants were also heritable in successive generations [168]. Another hormone DELLA is a negative regulator of gibberellin signaling. the loss-of-function mutations in *SlDELLA* exhibited high gibberellin sensitivity and a parthenocarpic phenotype [169,170]. All these findings suggested that the CRISPR/Cas9 system enhanced parthenocarpy in tomato plants. Other members of Solanaceae, such as peppers and eggplants, can also be improved by deploying the genome editing tool CRISPR/Cas9. Also, the overexpression of *ZmWRKY106* enhances drought and heat tolerance in transgenic maize plants by regulating the expression of stress-related genes, reducing ROS content, and by increasing the activities of antioxidant enzymes [171]. Heat shock proteins (HSPs) are molecular chaperones involved in cellular survival by transporting, folding, and degrading other proteins under heat stress [172]. The overexpression of HSP70 genes conferred increased resistance to abiotic stresses including high-temperature stress [173,174]. Moreover, the overexpression of HSP40 enhanced thermo-tolerance in transgenic Arabidopsis [175].

## 6. CRISPR/Cas9 for Mitigating the Impact of Cold Stress

In recent years, climate change has become the core problem threatening global food security [176]. In addition to heat waves, extremely cold temperatures have also been recorded in different ecological regions of the world [177]. Cold- or low-temperature stress may be divided into freezing stress (<0 °C) and chilling stress (0–15 °C), which adversely affects crop growth and production [178,179,180]. Excessive cold temperatures halt plant growth as it causes mechanical injury and dysfunction of metabolic activities [181]. Cold stress poses negative effects on the biochemical, physiological, and molecular activities of plants during their growth and development. Cold exposure, especially during winter, severely affects the photosynthetic potential and the plant anatomy [182,183]. Cold stress during the seedling stage may lead to poor germination and emergence. Prolonged exposures causes stunted growth, leaf chlorosis, poor source–sink relations, and nutrient localization [184]. The major impact of cold stress in plants is membrane rigidification, which aggravates other downstream processes in response to cold stress. Moreover, it disturbs the stability of the protein and expression, as well as impairs the activities of several enzymes including ROS-scavenging enzymes. Resultantly, the photosynthetic capacity of plant cells is questioned along with membrane damage and the formation of secondary structures in RNA that restrict its expression [185]. Low temperatures can injure crop species affecting their growth, productivity, and survival [186]. 

Various physiological and biochemical processes in plants are regulated by proline-rich proteins involved in growth and stress tolerance in plants. The knockout of *OsPRP1* (encodes proline-rich protein) by CRISPR/Cas9 enhanced the cold tolerance ability in rice [187]. In addition, the CRISPR/Cas9-mediated knockout of *OsMYB30*, characterized as a cold-responsive gene in rice, exhibited a higher cold tolerance than that of wild-type rice [104]. The CRISPR/Cas9-mediated ΔAtcbf single, double, and triple mutants in Arabidopsis elucidated that three tandemly arranged CCAAT-binding factor (CBF) genes such as *CBF1, CBF2*, and *CBF3* have been involved in cold acclimatization. The cold-acclimated Atcbf triple mutants exhibited a highly sensitive response under cold stress compared to that of the wild type. Under prolonged exposures to chilling temperatures, the expression of *CBF* genes was suddenly enhanced. Resultantly, CBF proteins activate the transcription of downstream cold-responsive genes to improve the freezing tolerance in plants [188]. The *CBF1* is the only cold-inducible gene in tomato plants and its increased expression resulted in salicylic acid and hydrogen peroxide-induced cold tolerance in tomato plants [189]. The *CBF1* mutants generated by the CRISPR/Cas9 exhibited greater electrolyte leakage and malondialdehyde (MDA) levels than wild-type plants in tomato plants, indicating that the knockout of *CBF1* can increase cold-stress-induced membrane damage [190]. Plant annexins and phospholipid-binding proteins are involved in the regulation of plant development and stress tolerance. The *OsAnn3*-knockout mutants developed by CRISPR/Cas9 showed enhanced relative electrical conductivity as compared to wild-type plants, which proved that *OsAnn3* can perform a role in cold tolerance in rice [191]. Several cis-regulatory elements in the rice promoter region *OsAnn5* are common promoter elements. However, some elements are unique to *OsAnn5*, including recognition sites for MYB, dehydration-responsive elements, and light-responsive elements, showing that several transcription factors regulate the expression of *OsAnn5* in rice. The elimination of *OsAnn5* function through CRISPR/Cas9 significantly increased the survival rates at the seedling stage under cold stress in rice demonstrating that *OsAnn5* regulates cold stress tolerance at the seedling stage in rice [192]. 

## 7. CRISPR for Improving Plant Tolerance to Climate Change

Climate change coupled with environmental pollution renders detrimental effects on the growth, development, phenology, and production potential of crop plants. Drastic changes in global environmental conditions have led to the development of climate-resilient phytoremediation methods. These approaches are of huge importance due to the current situation of the environmental crisis. Soil contamination with heavy metals and metalloids is one of the major harmful effects of environmental pollution worldwide. Some of the metal ions are carcinogenic pollutants with a long half-life and are non-degradable in the environment. Therefore, enhancing the adaptive potential of plants to the changing environmental conditions is a major concern regarding phytoremediation practice. Genome modification using artificial nucleases has the potential to enhance phytoremediation.

Recently, the CRISPR-Cas9-based gene editing approach has been extensively used for the phytoremediation of heavy metals. These modifications facilitate to control and stabilize the harmful effects of environmental pollutants on various crop plants. CRISPR-Cas9-based gene editing offers exciting options for photo technologies such as phytoremediation [193]. Several phytoremediators have been sequenced such as *Thlaspi caerulescens* (hyper-accumulator for Ni, Zn, and Cd), *Arabidopsis halleri* (hyper-accumulator for Zn and Cd), *Hirschfeldia incana* (for controlling Pb), *Brassica juncea*, and *Pteris vittata* [194,195,196,197]. Phytoremediation is the most effective, environmentally friendly, and cost-effective approach for the remediation of toxic metals and plant pollutants. Gene editing has the potential to modify the efficacy of phytoremediation for metal uptake, metal transport, and sequestration. Many genes including metal transporter, a metal chelator, phytochelatin, and metallothionein have been transferred to plants to enhance metal uptake and sequestration. For instance, the CRISPR-Cas9-based genome modification in a metal transporter gene *OsNRAMP5* resulted in low Cd-accumulation without affecting the yield in indica rice [105]. Moreover, transgenic plants developed by CRISPR-Cas9 genome editing demonstrated an increased ability to tolerate, detoxify, or accumulate heavy metals thereby promoting phytoremediation [198]. The overexpression of metallothioneins encoding genes (*MT1*, *MT2*, and *MTA1*) led to the increased potential of accumulating Cu, Zn, and Cd in Arabidopsis and tobacco [199,200]. Similarly, the overexpression of ATP Sulfurylase and Selenocysteine Methyltransferase gene in *B. juncea* led to enhanced tolerance towards Selenium [201]. The expression of *BcMT1* and *BcMT2* metallothionein genes from *B. campestris* to Arabidopsis resulted in increased tolerance to Cu and Cd [202]. CRISPR has opened the way of phytoremediation for many plants, such as maize and poplar, which were considered capable but not yet investigated due to the complex architecture of their genome. 

## 8. CRISPR for Improving Plant Tolerance to Herbicides and Heavy Metals

Heavy metals (As, Ni, Mn, Co, Cu, Zn, etc.) have been accumulated in soils due to several anthropogenic activities which exert negative impacts on plant growth by disturbing the cellular membranes, photosynthetic ability, and cellular respiration that ultimately limits crop productivity [203,204]. Moreover, heavy metals produce hydrogen peroxide (H_2_O_2_), free radicals such as hydroxyl radicals (OH), and superoxide radicals (O^−^_2_), which lead to oxidative stress [205]. In addition, the continuous application of herbicides used for eradicating weeds has produced herbicide resistance in several plants [206,207]. 

Several genes play a role in improving the tolerance to heavy metals in pants [208]. For instance, γ-glutamyl acyltransferase mutants expressed defensive properties against heavy metal toxicity, due to enhanced accumulation of glutathione. Hence, the development of mutants using CRISPR-Cas9 would be beneficial to cope with heavy metal stress in plants. Recently, *oxp1* CRISPR-mediated mutants in Arabidopsis showed increased resistance to Cadmium (Cd) [209]. In rice, several transporter genes (*OsNramp1*, *OsCd1*, and *OsNramp5)* are involved in the absorption of Cd by the roots [210]. Manipulation in the expression of these genes through CRISPR/Cas9 has resulted in minimizing the concentration of Cd in rice. Mutants of *OsLCT1* and *OsNramp5* generated through CRISPR/Cas9 has resulted in reduced levels of Cd in rice [105]. Likewise, *OsARM1* regulates the expression of Arsenic (As) linked genes in rice crops. The knock-out mutants of the *OSARM1* by CRISPR produced tolerance to As [209]. The *OsHAK1* gene controls the uptake and translocation of Cesium (Cs^+^) in rice. The CRISPR-Cas system was deployed to reduce the uptake of radioactive Cs by the rice plants. The knock-out mutants of *OsHAK1* exhibited a significant reduction in 137 Cs^+^ content levels in roots [106]. The *OsPRX2* is known to limit ROS production under K^+^ limiting conditions. The overexpression of *Os-PRX2* produced K^+^ deficiency tolerance by closing the stomata in rice [211]. The CRISPR/Cas9-mediated knock-out mutants in *OsARM1* expressed an improved tolerance to arsenic in rice plants [107]. 

The development of herbicide tolerance in crop plants is one of the major targets for increasing crop production. Currently, genome editing based on the CRISPR-Cas9 system has been used to develop herbicide-tolerant crops as an effective weed control strategy [212,213]. The recombination of acetolactate synthase generated using CRISPR/Cas9 produced herbicide resistance rice [110]. CRISPR/Cas9-based mutants in the *ALS* gene significantly enhanced herbicide tolerance in watermelons [111]. In addition, Herbicide resistant maize plants were developed using the same approach targeting *ALS1* and ALS2 genes [112]. Herbicide tolerance traits have been incorporated in rice by exploiting CRISPR-based editing in *OsALS1* [108,109]. The knock-out mutants in the *OsALS* gene of rice depicted strong herbicide tolerance potential by conferring resistance to imazapic and imazethapyr [109]. Recently, the CRISPR-Cas9-based targeted mutations in EPSPS (5-Enolpyruvylshikimate-3-phosphate synthase, PDS (phytoene desaturase), and ALS [37] conferred herbicide resistance in tomato plants [214]. 

## 9. Conclusions and Future Direction

Traditional crop improvement approaches, including molecular breeding, mutagenesis, and transgenics are time-consuming and expensive. Additionally, they are not specific in bringing intended change to crop plants. For example, molecular breeding is limited by species-specific barriers and is often inflicted by linkage drag, which brings with it many unwanted characteristics. The elimination of these so-called unwanted genomic chunks requires intensive backcrossing, which makes the procedure of developing new varieties quite challenging. On the other hand, genetic transformation allows the engineering of those traits that do not even exist in the plant gene pool. However, the use of GM crops has become so controversial that many countries in the world have completely banned the cultivation of GM crops. Consequently, the power of the “gene revolution” faded away before delivering. The advent of new genome editing tools such as CRISPR offers hope to address the issues associated with GM crops. If the selectable marker as well as the gene coding for Cas9 are removed from the plant genome, it would become similar to the one developed by non-genetic engineering tools. Therefore, an increasing number of countries are allowing the cultivation of transgene-free genetically edited crops. However, the major limitation to the application of CRISPR technology to improving field crops would be the scarcity of functionally characterized gene(s) involved in the agronomic traits [25]. The scarcity of validated targets would be one of the major bottlenecks in unlocking the CRISPR potential for developing climate-smart stress-resilient crops. Nevertheless, we have seen that CRISPR is being increasingly employed in field crops to help address climate issues. The development of abiotic stress-tolerant and heavy metal stress-tolerant plants through the manipulation of *cis-*, and *trans*-regulatory elements, resistance (*R*) genes, and susceptibility (*S*) genes will allow their open-field cultivation as genome-edited plants do not differ genetically from their unedited counterparts except for the desired genetic change at a specific location on the genome. Using multiplex genome editing would allow for the development of genome-edited crops engineered for tolerance against multiple traits in a single transformation event. Therefore, it is expected that genome editing will become the technology of choice for developing desired genetically and epigenetically [215,216] biotech crops for different purposes particularly to address the food shortage problems as well as to fight climate change more effectively. 

## Data Availability

Not applicable.
